# Association of *ATP1A1* gene polymorphism with thermotolerance in Tharparkar and Vrindavani cattle

**DOI:** 10.14202/vetworld.2015.892-897

**Published:** 2015-07-21

**Authors:** Neeraj Kashyap, Pushpendra Kumar, Bharti Deshmukh, Sandip Bhat, Amit Kumar, Anuj Chauhan, Bharat Bhushan, Gyanendra Singh, Deepak Sharma

**Affiliations:** 1 Division of Animal Genetics, Indian Veterinary Research Institute, Izatnagar, Bareilly, Uttar Pradesh, India; 2 Department of Animal Genetics and Breeding, Govind Ballabh Pant University of Agriculture and Technology, Pantnagar, Uttarakhand, India; 3 Division of Physiology and Climatology, Indian Veterinary Research Institute, Izatnagar, Bareilly, Uttar Pradesh, India

**Keywords:** *ATP1A1* gene, cattle, polymorphism, thermotolerance, Tharparkar, Vrindavani

## Abstract

**Aim:**

One of the major biochemical aspects of thermoregulation is equilibrium of ion gradient across biological membranes. Na^+^/K^+^-ATPase, a member of P type-ATPase family, is a major contributor to the mechanism that actively controls cross-membrane ion gradient. Thus, we examined *ATP1A1* gene that encodes alpha-1 chain of Na^+^/K^+^-ATPase, for genetic polymorphisms.

**Materials and Methods:**

A total of 100 Vrindavani (composite cross strain of Hariana x Holstein-Friesian/Brown Swiss/Jersey) and 64 Tharparkar (indigenous) cattle were screened for genetic polymorphism in *ATP1A1* gene, using polymerase chain reaction single-strand conformation polymorphism and DNA sequencing. For association studies, rectal temperature (RT) and respiration rate (RR) of all animals were recorded twice daily for 3 seasons.

**Results:**

A SNP (C2789A) was identified in exon 17 of *ATP1A1* gene. Three genotypes namely CC, CA, and AA were observed in both, Vrindavani and Tharparkar cattle. The gene frequencies in Tharparkar and Vrindavani for allele A were 0.51 and 0.48, and for allele C were 0.49 and 0.52, respectively, which remained at intermediate range. Association study of genotypes with RT and RR in both cattle population revealed that the animals with genotype CC exhibited significantly lower RT and higher heat tolerance coefficient than CA and AA genotypes.

**Conclusion:**

Differential thermoregulation between different genotypes of *ATP1A1* gene indicate that the *ATP1A1* gene could be potentially contributing to thermotolerance in both, Tharparkar, an indigenous breed and Vrindavani, a composite crossbred cattle.

## Introduction

Heat production and heat loss have a delicate balance, which is maintained by certain thermoregulatory mechanisms in response to ambient temperature and humidity combinations. When the heat loss is overrun by heat gain, the homeostatic mechanism triggers the thermoregulatory response of animal to maintain the body temperature within normal range. During heat stress, increase in the core body temperature due to failure of homeostatic mechanism reduces productivity of the animals below their original genetic potential in growth [1], milk production [2-4], milk constituents, [5,6] and reproduction [7-10].

The genes that contribute to either heat production or heat loss mechanisms are related to the tolerance against heat; along with certain biochemical and molecular processes that protect against cell injury caused by excessive heat accumulation. Many of the candidate genes responding to thermal stress have been identified using microarray assays or expression profiling [11]. Studies have been carried out to identify allelic variants in some genes that confer heat tolerance in cattle, such as slick hair gene [12], *ATP1B2* gene [13], *HSP70A1A* [14,15], *HSP90AB1* [16], etc. One of those genes is *ATP1A1*, which encodes for the α1 chain of Na^+^/K^+^-ATPase that contains the catalytic unit of the enzyme. The Na^+^/K^+^-ATPase is a membrane bound active transport system responsible for maintaining the low internal Na^+^ and high internal K^+^ across the plasma membrane that is typical to most animal cells. Na^+^/K^+^-ATPase alpha chain is expressed in all tissues predominantly in peripheral nerves and in erythrocytes [17]. The importance of Na^+^/K^+^-ATPase in basal metabolism can be highlighted by the fact that it consumes 19-28% of total ATP production in mammalian cells at rest, to actively transport 3 Na^+^ of and 2 K^+^ into the cell [18].

Na^+^/K^+^-ATPase activity has also been reported to affect blood pressure regulation [19] and osmotic stress management in saline water fish [20]. Genetic variants of the *ATP1A1* gene have been suggested to be involved in the salt hypertension in Dahl rats [21]; feed intake in cattle [22]; and essential hypertension in humans [23], all of which may evidently affect heat production, heat loss, and water mineral balance. Further, Yang [24] found Na^+^/K^+^-ATPase activity to be associated with heat resistance ability with a high heritability of 0.53 in cattle. It has also been shown that certain genotypic variants of different Na^+^/K^+^-ATPase subunits have different heat tolerance in cows [25-28]. A differential expression of *ATP1A1* in different seasons [29] is also suggestive of the gene to have a certain part in animals’ response to environmental attributes.

It has been established that Indian breeds of cattle are better able to regulate their body temperature in response to heat stress than the exotic breeds of cattle [30,31]. Further, increase in exotic blood level in crossbreds leads to depression of production potential during heat stress [3,32]. Tharparkar cattle have evolved in the hot arid western region of India and believed to have better thermotolerance, whereas Vrindavani strain has been developed by the composite cross of various taurine breeds with Hariana breed of cattle. Therefore, the present study was undertaken to study the polymorphism of *ATP1A1* gene and its association with thermoregulation in Tharparkar and Vrindavani cattle.

## Materials and Methods

### Ethical approval

The experiment was prior approved by the Animal Ethics Committee of the Institute constituted as per the article number 13 of the CPCSEA rules laid down by Government of India and conducted following the code of ethics for animal experimentation.

### Experimental animals

A total of 164 adult female cattle, comprising 100 Vrindavani crossbred (Hariana × Holstein-Friesian/Brown Swiss/Jersey) and 64 Tharparkar were included under present investigation maintained at Cattle and Buffalo Farm, Indian Veterinary Research Institute, Izatnagar, Bareilly (UP), India. About 10 ml of venous blood was collected from each animal under sterile conditions and genomic DNA was isolated by phenol: Chloroform extraction method [33].

### Genotyping

A set of primers (forward 5’-ACAAACAAAAGGGTCACAACAT-3’ and reverse 5’-CTTACCCTAGATCCTGGCTCAT-3’) reported by Liu, *et al*. [26] was used for amplification of 301 bp fragment of *ATP1A1* gene spanning around exon 17. Reaction mixture of 25 ml for polymerase chain reaction (PCR) was prepared containing 15.7 ml of nuclease free water, 2.5 ml ×10 Taq buffer, 2 ml of MgCl_2_ (25 mM), 1 ml (50 ng) genomic DNA, 1 ml of both forward and reverse primers (10 pM/ml) each, 0.8 ml of dNTPs mix (10 mM), and 1 ml of Taq dna polymerase (1 U/ml). The program for thermal cycling included an initial denaturation step at 95°C for 5 min, followed by 30 cycles of denaturation at 95°C for 60 s, annealing at 60°C for 45 s, extension at 72°C for 60 s, and final extension at 72°C for 10 min.

The 5 ml of the PCR products was added to 15 ml formamide dye (95% v/v Formamide, 4% v/v 0.5 M Ethylenediaminetetraacetic acid [EDTA], 0.025% w/v bromophenol blue, and 0.025% w/v xylene cyanol) and mixed properly followed by denaturation at 95°C for 10 min then snap chilling on ice for 15 min, to form different single stranded DNA confirmations. Resolution of genotypes was carried out by electrophoresis using ×1 TBE (45 mM Tris-borate/mM EDTA) at 4°C on 12% polyacrylamide gel (acrylamide/bisacrylamide 49:1, v/v) for 18 h at 130 V. PCR-single strand conformation polymorphism (PCR-SSCP) banding patterns were detected by silver staining according to Bassam, *et al*. [34]. The representative samples of different PCR-SSCP patterns were purified with QIA quick^®^ Gel extraction kit (QIAGEN), cloned in pGEM^®^-T easy vector (Promega), and sequenced by the Sanger’s dideoxy chain termination sequencing method from both forward and reverse directions by primers for *ATP1A1* gene.

### Environmental and physiological parameters

Daily mean temperature was taken as the average of maximum and minimum temperatures of the day, recorded over 3 consecutive days of each season, i.e., winter (January), spring (March), and summer (May). The temperature humidity index (THI) was derived from a combination of wet- and dry-bulb air temperatures recorded for each day and expressed as THI = 0.72 (T_W_ + T_D_) + 40.6 [35] where, T_W_ is wet-bulb air temperature (in°C) and T_D_ is dry-bulb air temperature (in°C).

Rectal temperature (RT) was measured in all animals twice daily, morning at 10:00 am, and afternoon at 2:00 pm, for 3 consecutive days in each season. During the same time, average respiration rate (ARR) was determined by monitoring flank movements of the animal for 1 min while resting. Heat tolerance coefficient (HTC) was calculated for each cow as an index of its heat tolerability. HTC and RR were measured in all 150 Vrindavani and 64 Tharparkar cows in all 3 test periods. Iberia heat tolerance test was used to determine HTC: HTC = 100-10 (ART-38.3) [36]; where, ART is the average of RT at 10:00 am and 3:00 pm of 3 consecutive days for each test period and 38.3 is the normal RT of cattle (in °C).

### Statistical analysis

The association of the polymorphism of the *ATP1A1* gene with thermoregulatory traits was analyzed using GLM procedure of SAS software package version 9.3, which uses the method of least squares to fit general linear models. The least squares mean of different RT (10 am, 2 pm, and average) and ARR were compared by model: Y_ijkl_ = µ + B_i_ + S_j_ + G_k_ + e_ijkl_, where, Y_ijkl_ is the observation of the trait; m is overall mean; B_i_ is the fixed effect of i^th^ breed; S_j_ is the effect of j^th^ season; G_k_ is the fixed effect of k^th^ genotype; and e_ijkl_ is random error.

## Results and Discussion

### Polymorphism in *ATP1A1* gene

**Figure-1:**
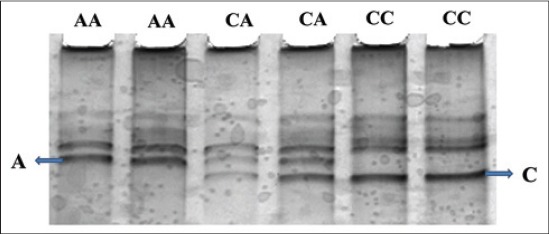
SSCP genotypes of amplified fragment covering exon 17 of *ATP1A1* gene.

**Figure-2:**
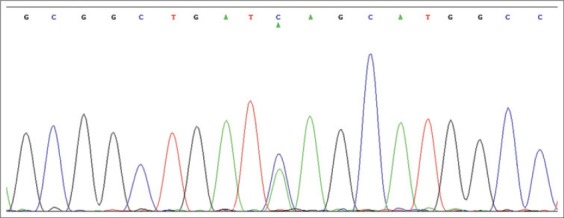
Sequencing chromatogram of heterozygote cattle.

A 301 bp fragment of *ATP1A1* gene encompassing exon 17 was amplified successfully with the used pair of primers. 3 genotypes (AA, AC, and CC) and 2 alleles namely A and C were evident on PCR-SSCP by different banding patterns (Figure-1). The nucleotide sequencing and analysis of these genotypes revealed that a SNP (C>A) was located at 29140575 base of *Bos taurus* chromosome 3 corresponding to 2789 base of *ATP1A1* mRNA (Figure-2). The identified SNP was a synonymous mutation. The sequences of allele A and C found in Tharparkar and Vrindavani were submitted to NCBI with accession number JX489364, JX489365, JX489366, and JX489367. The genotype frequencies for AA, AC, and CC genotypes were 0.24, 0.47, and 0.29 while 0.31, 0.39, and 0.30 in Vrindavani and Tharparkar, respectively. The gene frequencies for allele A and C were 0.51 and 0.49 and 0.52 and 0.48 in Vrindavani and Tharparkar, respectively. Frequency of heterozygotes was found much more than either the homozygotes in both cattle population. Though the frequencies of alleles did not vary much between Tharparkar and Vrindavani, they were found differing from that of the Chinese Holstein cattle where allele C was found most frequent (0.86) [26].

### Environmental parameters

For winter, spring, and summer, the average daily temperature (°C) was 10.83, 22.67, and 33.16, respectively, while RH (%) was recorded to be 70, 41, and 33.33, respectively. McDowell, *et al*. [35] suggested that the THI could be used as a better indicator of thermal climatic conditions. The THI values were lowest for winter (52.72), followed by spring (68.25), and highest for summer (84.34). Considering threshold for heat stress on cattle at THI value of 72 [37], the THI values for test period of summer season exceeded the comfort zone of THI for dairy cattle, thus imposing heat stress on animals. However, the THI values for winter and spring were below the critical THI values and remained within the comfort zone for cattle.

### Thermoregulatory parameters

In both cattle populations, the effect of genotypes was found statistically significant (p≤0.01) for 10 am RT (RT_M_), 2 pm RT (RT_A_), average RT (ART), and HTC; while it was statistically non-significant for ARR. The CC genotype accounted for lowest values for RT_M_, RT_A_, ART, and highest value for HTC while there was no significant difference between genotype AA and CA for any of these parameters. For ARR, the difference was not statistically significant between genotype AA, AC, and CC in Tharparkar; while in Vrindavani, genotype CC was statistically different (p≤0.01) and lower from genotype AA and CA (Table-1). The effect of season was found significant, with highest RT_M_, RT_A_, ART and RR but lowest HTC values in summer in both cattle populations (Table- 2).

**Table-1:** Effect of the genotype at exon 17 of *ATP1A1* gene on thermotolerance in cattle.

Genotype	RT_M_	RT_A_	ART	ARR	HTC
Tharparkar					
CC	38.77^a^±0.03	38.56^a^±0.03	38.26^a^±0.04	15.62^a^±0.14	97.82^a^±0.33
CA	38.88^b^±0.03	38.65^b^±0.02	38.42^b^±0.03	15.98^a^±0.12	96.47^b^±0.29
AA	38.93^b^±0.03	38.71^b^±0.03	38.48^b^±0.03	16.05^a^±0.14	95.91^b^±0.32
Vrindavani					
CC	38.25^a^±0.02	38.70^a^±0.02	38.48^a^±0.02	15.56^a^±0.09	98.23^a^±0.19
CA	38.34^b^±0.02	38.82^b^±0.02	38.58^b^±0.02	16.12^b^±0.07	97.18^b^±0.15
AA	38.34^b^±0.02	38.82^b^±0.02	38.58^b^±0.02	15.98^b^±0.10	97.23^b^±0.21

Different superscript in same column indicate significant differences (p≤0.01), RT_M_=Rectal temperature at 10 am, RT_A_=Rectal temperature at 2 pm, ART=Average rectal temperature, ARR=Average respiration rate, HTC=Heat tolerance coefficient

**Table-2:** Effect of season on thermotolerance in cattle.

Season	RT_M_	RT_A_	ART	ARR	HTC
Tharparkar					
Winter	38.17^a^±0.03	38.61^a^±0.03	38.39^a^±0.03	14.86^a^±0.14	99.11^a^±0.31
Spring	38.37^b^±0.03	38.85^b^±0.03	38.61^b^±0.03	15.51^b^±0.14	96.90^b^±0.31
Summer	38.63^c^±0.03	39.13^c^±0.03	38.88^c^±0.03	17.28^c^±0.14	94.19^c^±0.31
Vrindavani					
Winter	37.93^a^±0.02	38.46^a^±0.02	38.20^a^±0.02	14.02^a^±0.08	101.01^a^±0.18
Spring	38.30^b^±0.02	38.73^b^±0.02	38.50^b^±0.02	15.22^b^±0.08	97.89^b^±0.18
Summer	38.70^c^±0.02	39.15^c^±0.02	38.93^c^±0.02	18.43^c^±0.08	93.74^c^±0.18

Different superscript in same column indicate significant differences (p≤0.01), RT_M_=Rectal temperature at 10 am; RT_A_=Rectal temperature at 2 pm, ART, Average rectal temperature, ARR=Average respiration rate; HTC=Heat tolerance coefficient

**Figure-3:**
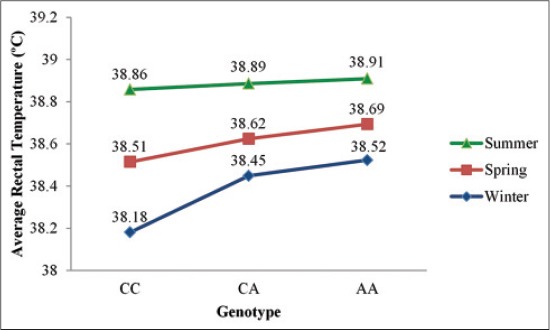
Average rectal temperature of different genotypes in Tharparkar.

**Figure-4:**
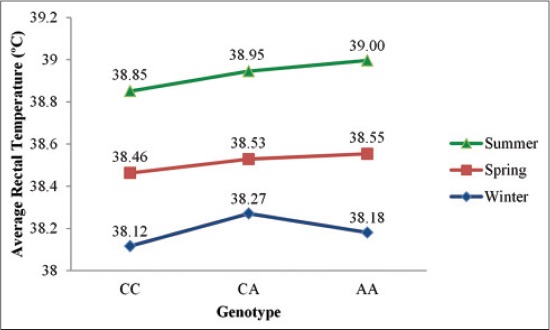
Average rectal temperature of different genotypes in Vrindavani.

The animals with genotype CC maintained the lowest ART followed by animals with genotype CA and AA in Tharparkar (Figure-3) as well as in Vrindavani (Figure-4) for each season. The consistency of the higher HTC in genotype CC through all seasons suggests probable differential heat production or heat loss between the genotypes. In the present study, higher thermotolerant effects of genotype CC in summer was in agreement to the findings of Liu, *et al*. [26] on Chinese Holstein cattle and extends their findings in zebu-taurine crossbreds and indigenous zebu cattle. However, in contrast to their findings the effect of the genotype in this study was not restricted to heat tolerance; rather it was contributing to thermal balance in all seasons.

## Conclusions

There are certain genes, controlling response to heat stress either by altering heat production and heat loss balance or by exerting cytoprotective actions. One of those genes conferring heat tolerance is *ATP1A1*, encoding for the α1 chain of Na^+^/K^+^-ATPase. The effects of genotypes for exon 17 of *ATP1A1* showed significant association with heat tolerance parameters. Genotype CC had a significantly (p≤0.01) lower HTC value, RT, and RR in both Tharparkar and Vrindavani, marking it as more efficiently thermoregulated genotype. Furthermore, genotype CC was associated with not only lower RT and RR in summer, but in all seasons, suggesting that genotype CC confers to overall low thermal balance, that is extended to be favorable in terms of heat tolerability in summer.

## Authors’ Contributions

NK: Research was done by this author as the part of his master’s degree thesis dissertation. PK: Designed the study and supervised the research as major advisor of NK. BD: Worked and collaborated in the standardization of protocols and compilation of the results reported in the manuscript as well as compilation of the manuscript. SB: Collaborated in the lab work and shared a lot of opinions regarding the work. AK and AC: Provided valuable suggestions regarding the design of the experiment and analysis of the data collected during research. BB: As a member of advisory committee, contributed in all aspects of the work and shared lab facilities to facilitate experiments. GS: As an expert of animal physiology and climatology, provided valuable suggestions and implementable ideas of recording physiological and climatological parameters. DS: Provided valuable suggestions regarding the conduct of the experiment and necessary timely support needed to complete the work. All authors read and approved the final manuscript.
